# On Apoplexy

**Published:** 1802-05

**Authors:** 


### *To the Editors of the Medical and Physical Journal.*

GENTLEMEN,

THE difpute which has lately been carried on through the medium of your Journal on the caufe of Apoplexy, although it appears to have primarily arifen from fome perfonal pique, yet as the fubject of it is a difeafe concerning which the medical world has not come to a final conclufion, it may in the end be productive of advantage.

I muft confefs it is my opinion that the general caufe is compreffion of the brain, either from over diftention of the veffels of the head or actual extravafation; except when fome of thofe agents have been applied, "which directly deftroy the "mobility of the nervous power, as the fumes arifing from "fermenting liquors, or burning charcoal, of mercury or lead, "and fome other metallic fubftances; opium, alcohol, and "fome other narcotic poifons."

I cannot

I cannot bring myſelf to think a meal of common food can act in a manner in the leaſt analagous to the agents above enumerated, as Mr. Crowfoot ſeems to think. We know that every thing which impedes the free paſſage of the blood through the lungs, likewiſe prevents the free return of it into the right ventricle of the heart, and a conſequent accumulation takes place in the veſſels of the head.

Now, a meal,* by diſtending the ſtomach, muſt neceſſarily prevent the diaphragm from deſcending, by which the tranſmiſſion of the blood through the lungs is impeded; that fluid collects on the right ſide of the heart, and the veſſels of the brain ſwelled out much beyond their natural circumference, may compreſs the medullary portion of that viſcus, or actually give way from over diſtenſion, and in either way produce the ſymtoms of Apoplexy.

The truth of the above Statement will be more obvious, when we conſider that at the time which this diſeaſe moſt generally occurs, a venous plethora exiſts in the ſyſtem, conſequently a ſlower return of blood from the head, which may be more or leſs in different perſons, the variety conſiſting in many local cauſes; and that when this pre-diſpoſition exiſts we are not to be ſurpriſed that a meal ſhould produce ſuch effects.

Concerning the propriety or impropriety of emetics in caſes of Apoplexy, I believe the fear of uſing them has rather ariſen from conjecture à priori, than from actual experience of their bad effects. Dr. Cullen in his Practice of Phyſic, makes no mention of any harm from their uſe in theſe caſes, as doubtleſs ſuch an accurate obſerver would have done if he had only heard of any, but acknowledges that his reaſon for not uſing them was the apprehenſion that they might impel the blood with too much force into the veſſels of the head. In fine, the only queſtion to be aſked is, whether the benefit of emptying the ſtomach, and conſequently of providing a more free paſſage of the blood from the head, overbalances the evil of increaſing the celerity of the circulation (which might certainly be obviated by a moderate bleeding previous to the exhibition of the emetic) in favour of which experience I think has decided.

When extravaſation has once taken place to a *conſiderable extent*, I much fear that remedies will be of little ſervice; but as apoplectic ſymptoms may be produced by ſimple diſtenſion of the veſſels of the brain, every mode which experience countenances ſhould be immediately had recourſe to, to remove the diſtenſion, or, if extravaſation has actually begun, to prevent any further progreſs of it. I am, &c.

H. H.

---

* Recollect, that it is immediately after a meal that Apoplexy very generally attacks the patient.

